# Online adaptive MR-guided radiotherapy: Conformity of contour adaptation for prostate cancer, rectal cancer and lymph node oligometastases among radiation therapists and radiation oncologists

**DOI:** 10.1016/j.tipsro.2022.08.004

**Published:** 2022-08-24

**Authors:** Marnix J.A. Rasing, Gonda G. Sikkes, Nicole G.P.M. Vissers, Alexis N.T.J. Kotte, Joske H. Boudewijn, Patricia A.H. Doornaert, Wietse S.C. Eppinga, Martijn Intven, Reijer H.A. Rutgers, Annick Scheeren, Louk M.W. Snoeren, Tiny B. Vlig, Jochem R.N. van der Voort van Zyp, Lisa M. Wijkhuizen, Peter S.N. van Rossum, Max Peters, Ina M. Jürgenliemk-Schulz

**Affiliations:** a Department of Radiation Oncology, University Medical Center Utrecht, Heidelberglaan 100, 3584 CX Utrecht, The Netherlands; b Radiotherapeutisch Instituut Friesland, Borniastraat 36, 8934 AD Leeuwarden, The Netherlands

**Keywords:** MR-guided radiotherapy, Image-guided radiation, MR-Linac, Conformity, Delineation, Adaptive

## Abstract

**Background and purpose:**

Online adaptive MR-guided treatment planning workflows facilitate daily contour adaptation to the actual anatomy. Allocating contour adaptation to radiation therapists (RTTs) instead of radiation oncologists (ROs) might allow for increasing workflow efficiency. This study investigates conformity of adapted target contours provided by dedicated RTTs and ROs.

**Materials and methods:**

In a simulated online procedure, 6 RTTs and 6 ROs recontoured targets and organs at risk (OAR) in prostate cancer (n = 2), rectal cancer (n = 2) and lymph node-oligometastases (n = 2) cases. RTTs gained contouring competence beforehand by following a specific in-house training program. For all target contours and the reference delineations volumetric differences were determined and Dice similarity coefficient (DSC), conformity index (CI) and generalized CI were calculated. Delineation time and –confidence were registered for targets and OAR. Impact of contour adaptation on treatment plan quality was investigated.

**Results:**

Delineation conformity was generally high with DSC, CI and generalized CI values in the range of 0.81–0.94, 0.87–0.95 and 0.63–0.85 for prostate cancer, rectal cancer and LN-oligometastasis, respectively. Target volumes were comparable for both, RTTs and ROs. Time needed and confidence in contour adaptation was comparable as well. Treatment plans derived with adapted contours did not violate dose volume constrains as used in clinical routine.

**Conclusion:**

After tumor site specific training, daily contour adaptations as needed in adaptive online radiotherapy workflows can be accurately performed by RTTs. Conformity of the derived contours is high and comparable to contours as provided by ROs.

## Highlights

• Contour adaptation conformity analyzed for LN-metastases, rectal- + prostate cancer.
• Contour adaptation conformity among RTTs and radiation oncologists is comparable.
• Role expansion of RTTs with daily contour adaptation impacts workflow efficiency.

## Introduction

In current radiotherapy routine, registration and interpretation of cone-beam CT (CBCT) information is commonly performed by radiation therapists (RTTs) [1], [2]. Subsequently, based on action levels, RTTs decides if the radiotherapy fraction can start, or if a medical physicist (MP) or radiation oncologist (RO) should be called for consultation. One step towards online adaptive radiotherapy was the introduction of a library-of-plans approach, which provides the ability to better tailor the dose to the target and away from surrounding organs by means of RTTs selecting the most fitting version from a library of pre-defined treatment plans [3], [4].

In the pretreatment phase organ-at-risk (OAR) delineations are often performed by RTTs [5]. Following adequate training, RTTs achieve a good concordance with OAR contoured by ROs [6]. Furthermore, evidence is published showing that trained RTTs are capable of delineating pre-treatment targets based on standardized contouring protocols [7], [8].

The MR-Linac Unity system of Elekta [9] and MRIdian system of Viewray [10] enable additional functionalities with online adaptive MR-guided radiotherapy (oMRgRT), which include daily MR scanning, image fusion with pre-treatment MR images, contour propagation based on pre-treatment delineations, delineation adjustments, planning, position verification, planning quality assessment and radiation delivery [11]. Interfraction variations can be decreased with daily online contouring and planning adaptations, providing opportunities for decreased PTV margins or dose escalation [9], [12]. A drawback for patients is the time-consuming nature of online workflows. Additionally, department logistics is challenging for these treatments due to the need for ROs, MPs and RTTs to be available during the online procedure. This study was performed with the MR-Linac Unity system, therefore we will refer to the corresponding adapt-to-shape (ATS) workflow [9].

Online contour adaptation is currently recognized as the most time-consuming part of the workflow. Moreover, contour adaptation is the part with the highest observer dependency [13]. Interobserver variability for target and OAR contours is persistent even when modern imaging modalities and joint contouring guidelines are used [14], [15]. Therefore, balancing between delineation time and contouring precision for every treatment fraction remains a challenge.

At our institute, efforts have been made to optimize the clinical efficiency of the ATS workflow including the transfer of online OAR contouring and selected treatment targets from ROs to RTTs [12], [16], with potential benefit for patients, multidisciplinary team and department logistics. This step was introduced for treatments of lymph node (LN) oligometastases in 2019, after a specific in-house developed training program for RTTs that provides RTTs with knowledge and skills necessary for adapting contours when applying ATS workflows on the MR-Linac.

The aim of this study was to quantify interobserver conformity for target contour adaptations among ROs and RTTs and their possible impact on MR-Linac treatment plans for patients with LN-oligometastases, rectal- and prostate cancer.

## Material and methods

All patients included in this study gave written informed consent for participation in the Momentum cohort (NCT04075305) and have been treated on the 1.5 T MR-Linac.

### Clinical workflow

Target and OAR contouring for pre-treatment planning is performed using a simulation-MRI. Contours are approved by a dedicated RO. The pre-treatment contours are deformably registered to the online MRI as performed on the MR-Linac for each fraction. During treatment, the respective pre-treatment reference MRI with target and organ contours, is visually assessable using an in-house developed software program Volumetool [17]. Supported by this information specifically trained RTTs perform online contour adaptations for GTV/CTV and OAR if necessary. Adaptations are performed inside a 2 cm ring structure around the PTV in order to support online treatment planning. A RO is always available on demand. The specific training encompasses knowledge of MR sequences and image registration, MR-guided CTV and OAR contouring guidelines, gaining offline contouring experience, followed by supervised on-site contouring (including ≥ 15 cases per tumor site) and ends with supervision on demand (Supplementary Table A).

### Data generation

For this study 6 patients were selected: with prostate cancer (n = 2), rectal cancer (n = 2), a single LN-oligometastasis (n = 1) and two nodal oligometastases (n = 1). On a yearly basis, around 160 prostate cancer-, 40 rectal cancer- and 70 LN-oligometastasis patients are treated at our department with an ATS workflow. In order to simulate the online procedure for research purposes, an educational environment in the planning software Monaco (Monaco Educational, Elekta AB, Sweden) was used for adapting contours from the pre-treatment MRI to the anatomical situation of the first online T2-weighted MRI.

In accordance with the online situation, the structures subject to adjustment were CTV in prostate cancer cases, CTV of mesorectum, presacral and elective nodal areas in rectal cancer cases, GTVs in LN cases and OAR inside the 2 cm ring around the PTV. For rectal cancer cases, a total CTV was manually generated by combining all sub-CTVs and no OAR had to be delineated. When delineating OAR, the outer contour was delineated. The pre-treatment imaging and -delineations were in parallel available in Volumetool and the participants were instructed to limit contour adaptation time to approximately 15 min. Six ROs and 6 RTTs participated, all of them regularly treat patients on the MR-Linac. The 6 RTTs gained full competence for the contouring part, 6–12 months prior to this study. At the time of study participation, all 6 RTTs performed the respective MR-Linac workflows entirely with supervision on demand. They performed their contouring training at different moments in time, resulting in some differences in clinical contouring experience. Some difference in experience also applies to the ROs, due to specializations in different tumor sites.

### Analysis

Participating ROs and RTTs registered the time required for contouring and filled in a confidence questionnaire on their individual contouring experience: not confident, uncertain of GTV/CTV, uncertain of OAR, rather confident or very confident. For all cases, the contour used clinically for the first online fraction was defined as reference contour. Countmaps of overlapping GTV and CTV-contours of all delineators were generated with voxel size 0.8 × 0.8 × 2 mm, in which interrater agreement per voxel is indicated by differences in color. We assessed volumetric differences in GTVs, CTVs and PTVs and calculated a Dice similarity coefficient (DSC) and conformity index (CI), also known as concordance index [5], [14], [18] for all study contours and the reference delineation in order to evaluate possible differences. Also, the generalized CI was determined [19], defined as the total of the common volumes between observer pairs divided by the total of the encompassing volumes between each observer pair. Conformity was measured for RTTs versus ROs, within RTTs, within ROs and for RTTs or ROs versus the reference delineation.

Subsequently, we analyzed if adequate target coverage and compliance with constraints for OAR were feasible for the newly contoured delineations, by generating new treatment plans for the delineations with the smallest and largest volume per case, using an offline ATS workflow. Delineation conformity of OAR was not assessed as this is already part of clinical routine in our institute for several years, but OAR were included in assessing delineation time and -confidence and treatment planning.

#### Statistical methods

Analyses were performed using SPSS version 25.0 (IBM Corp, IBM SPSS Statistics for Windows, Armonk, NY). Boxplots were generated to present DSC and CI among RTTs and ROs for GTV/CTV and PTV structures per case, with medians, interquartile ranges (IQR), complete ranges and outliers. Mann-Whitney *U* tests were used to test for significant differences in DSC, CI and delineation time. We tested for significant differences in conformity levels in two ways. Firstly, DSC and CI of the group RTTs versus the reference delineations were compared to DSC and CI of the group ROs versus the reference delineations. Secondly, DSC and CI within the group of RTTs were compared to DSC and CI within the group of ROs. The association between conformity and delineation time was analyzed using linear regression analysis after dichotomization. The association between conformity and delineation confidence was determined using binary logistic regression analyses. Since delineation time and -confidence were measured per session, the case with two LN targets was not included in these analyses.

## Results

**Fig. 1:**
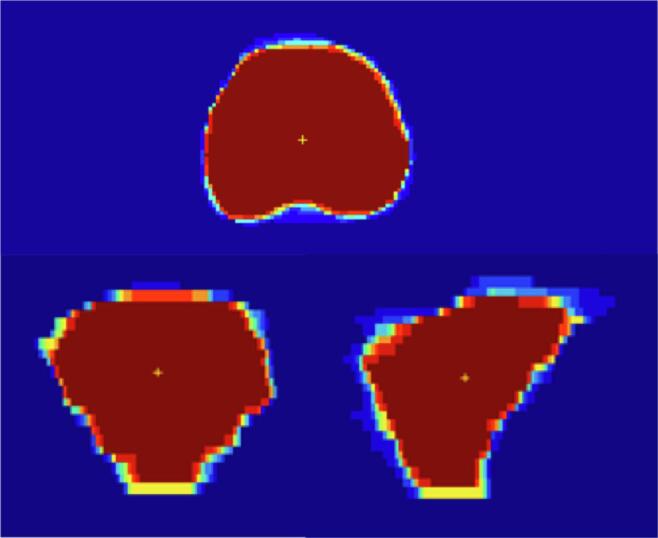
Countmap of contours derived for a prostate case to illustrate interrater conformity in an axial view (upper contour), coronal view (lower left contour) and sagittal view (lower right contour). The colors represent interrater agreement per voxel. Voxel size: 0.8 × 0.8 × 2 mm. Dark red = 11 delineators, bright orange = 9 delineators, yellow = 7 delineators, light blue = 4 delineators, dark blue = 0 delineators.

Table 1 presents volumetric parameters of all target volumes from the total group of 12 delineators and from the 6 RTTs and 6 ROs separately. Across the total group as well as within both subgroups, the derived volumes for all target sites were quite comparable and not systematically larger or smaller. A visual impression of the contour variations using countmaps of overlapping delineations is presented in Fig. 1 and Supplementary Fig. A.1 and A.2 for a prostate, LN and rectum case, respectively.

**Table 1:** Volumetric parameters for GTV, CTV and PTV in mL.

	Prostate 1		Prostate 2		LN single		LN multiple				Rectum 1		Rectum 2	
	CTV	PTV	CTV	PTV	GTV	PTV	GTV1	PTV1	GTV2	PTV2	CTV	PTV	CTV	PTV
Reference*volume	48.4	94.1	39.6	81.6	0.5	2.3	0.5	2.4	0.7	2.8	378	605.2	439.2	718.4

Radiotherapists														
Mean vol.	47.5	95.8	37.4	79	0.4	1.9	0.5	2.3	0.7	2.7	369	581.9	441.3	704.3
SD	1	2.6	2.6	5.1	0.1	0.3	0.1	0.3	0.1	0.3	14	18.9	14.7	20.4
Minimum	46.2	92.2	33.5	70.9	0.3	1.3	0.3	1.7	0.5	2.1	351.4	555.7	417.9	676.2
Maximum	49	99.7	40.3	84	0.4	2.1	0.6	2.6	0.8	3.1	392.3	612.5	460.4	735.6

**Radiation oncologists**														
Mean vol.	46.9	97.4	38.1	82.2	0.3	1.7	0.4	2.1	0.6	2.4	371.6	586.9	446.3	713
SD	2	5.4	2.2	4.3	0.1	0.2	0.1	0.2	0.1	0.3	11.7	14.3	16	18.7
Minimum	45.9	94.2	35.6	77.8	0.2	1.4	0.4	1.9	0.4	2	354.8	563.4	426.7	687.6
Maximum	50.4	107.1	41.4	88.7	0.4	1.9	0.5	2.3	0.7	2.7	384	598.4	469.4	740.2

**Whole group**														
Mean vol.	47.2	96.5	37.7	80.5	0.4	1.8	0.5	2.2	0.6	2.6	370.2	584.2	443.5	708.3
SD	1.4	4	2.3	4.8	0.1	0.3	0.1	0.2	0.1	0.3	12.4	16.4	14.8	19.2
Minimum	45.9	92.2	33.5	70.9	0.2	1.3	0.3	1.7	0.4	2	351.4	555.7	417.9	676.2
Maximum	50.4	107.1	41.4	88.7	0.5	2.1	0.6	2.6	0.8	3.1	392.3	612.5	469.4	740.2

Abbreviations: GTV, Gross Tumor Volume; CTV, Clinical Target Volume; PTV, Planning Target Volume; LN, lymph node; vol., volume; SD, standard deviation.

* First online delineation.

Table 2 provides DSC and CI-values for the different target sites and for the following groups of delineators: RTTs versus ROs, within RTTs, within ROs, RTTs or ROs versus reference contours.

**Table 2:** Dice similarity coefficient and conformity index values of GTV and CTV.

	Prostate 1	Prostate 2	LN single	LN multiple		Rectum 1	Rectum 2
	CTV	CTV	GTV	GTV1	GTV2	CTV	CTV
**Radiotherapists compared to Radiation oncologists**							
DSC							
Mean	0.93	0.91	0.8	0.79	0.79	0.94	0.94
SD	0.03	0.02	0.07	0.08	0.08	0.02	0.01
CI							
Mean	0.86	0.83	0.67	0.67	0.66	0.89	0.89
SD	0.05	0.03	0.09	0.11	0.11	0.03	0.02

**Radiotherapists compared to other Radiotherapists**							
DSC							
Mean	0.94	0.91	0.84	0.76	0.79	0.94	0.94
SD	0.01	0.02	0.03	0.11	0.11	0.02	0.01
CI							
Mean	0.89	0.84	0.72	0.63	0.67	0.88	0.88
SD	0.01	0.03	0.04	0.15	0.14	0.03	0.02

**Radiation oncologists compared to other Radiation oncologists**							
DSC							
Mean	0.91	0.9	0.79	0.83	0.81	0.95	0.94
SD	0.04	0.02	0.07	0.03	0.04	0.01	0.01
CI							
Mean	0.84	0.82	0.66	0.71	0.68	0.9	0.89
SD	0.07	0.04	0.1	0.04	0.05	0.01	0.01

Radiotherapists compared to Reference*							
DSC							
Mean	0.94	0.91	0.85	0.83	0.82	0.94	0.93
SD	0.01	0.02	0.04	0.11	0.11	0.01	0
CI							
Mean	0.89	0.83	0.74	0.71	0.71	0.89	0.87
SD	0.01	0.03	0.05	0.15	0.14	0.02	0.01

Radiation oncologists compared to Reference*							
DSC							
Mean	0.92	0.89	0.77	0.85	0.8	0.94	0.93
SD	0.03	0.02	0.09	0.03	0.05	0.01	0
CI							
Mean	0.85	0.81	0.63	0.74	0.67	0.88	0.87
SD	0.05	0.03	0.11	0.04	0.07	0.01	0.01

**Generalized conformity index (CI)**							
Generalized CI							
	0.87	0.83	0.68	0.67	0.67	0.89	0.88

Abbreviations: GTV, Gross Tumor Volume; CTV, Clinical Target Volume; LN, lymph node; DSC, Dice similarity index; SD, standard deviation; CI, conformity index.

* First online delineation.

In prostate cancer cases, mean DSC varied between 0.89 and 0.94, mean CI between 0.81 and 0.89 and generalized CI between 0.83 and 0.87. In rectal cancer cases, mean DSC, CI and generalized CI-values varied between 0.93 and 0.95, 0.87–0.90 and 0.88–0.89 and in LN-oligometastasis cases between 0.76 and 0.85, 0.63–0.74 and 0.67–0.68 respectively.

**Fig. 2:**
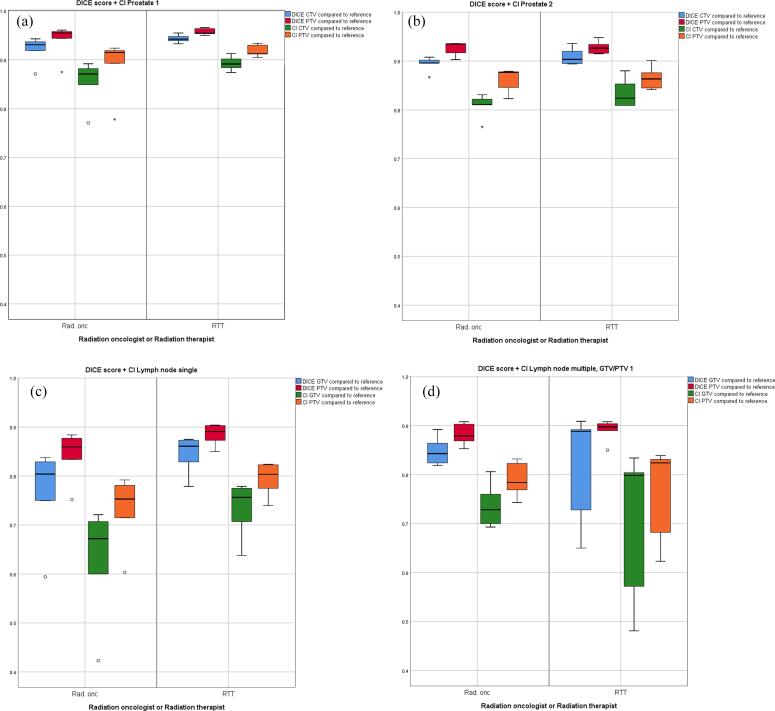

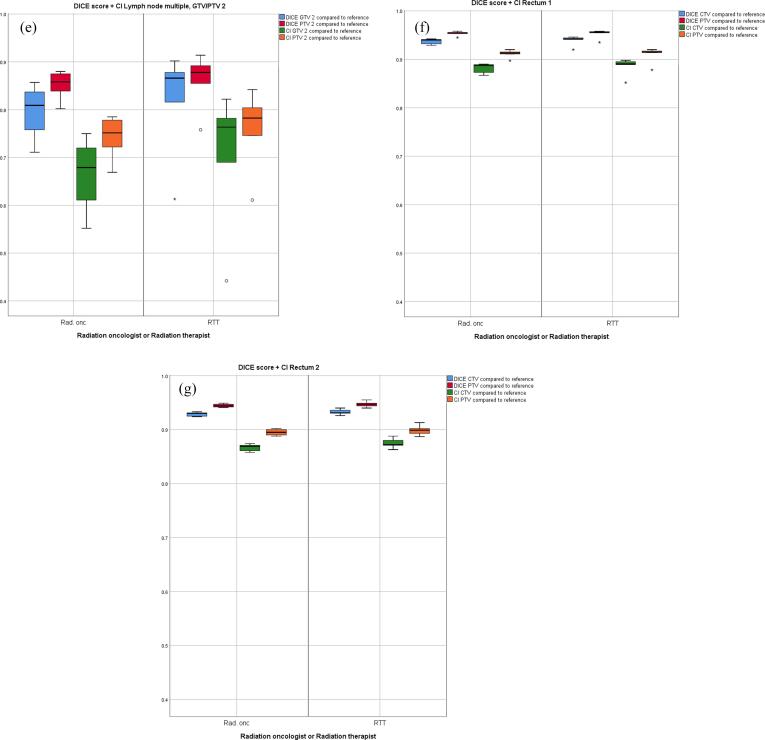
Boxplots indicating the Dice similarity coefficient and conformity index for both radiotherapist and radiation oncologists versus the reference delineation for GTV/CTV and PTV structures per case: prostate 1 (a), prostate 2 (b), lymph node single (c), lymph node multiple (d,e), rectum 1 (f), rectum 2 (g). Abbreviations: GTV, Gross Tumor Volume; CTV, Clinical Target Volume; PTV, Planning Target Volume; SC, similarity index; CI, conformity index; RTT, radiotherapist; Rad. onc, radiation oncologist. Medians with interquartile ranges (IQR) and complete ranges are illustrated. The circles represent outliers, defined as outside the 1.5 * IQR and the asterisks represent extreme outliers, defined as outside the 3 * IQR.

The DSC scores are structurally somewhat higher compared to the CI-values. When testing for significant differences in DSC and CI of the group RTTs versus the reference delineations compared to DSC and CI of the group ROs versus the reference delineations, only for the CTV in prostate case 1 a marginally significant difference was found (*p* = 0.045 for both DSC and CI). No significant differences were found when DSC and CI within the group of RTTs were compared to DSC and CI within the group of ROs. Fig. 2 provides a graphic comparison of conformity between RTTs and ROs, with boxplots of DSC and CI for GTV/CTV and PTV structures for all cases compared to the reference delineation. Of interest are the lower values of conformity and larger interquartile ranges for LN cases, showing an increased distribution in conformity levels between delineators.

Delineation time is presented in Table 3. No significant differences were found in delineation time for RTTs compared with ROs. When all 6 cases were pooled, more delineation time was significantly associated with increased conformity for the total group as well as within RTTs and within ROs (Supplementary Table B, Supplementary Fig. B). This effect was not observed when analyzing the cases individually.

**Table 3:** Delineation time (minutes: seconds).

	Prostate 1	Prostate 2	LN single	LN multiple	Rectum 1	Rectum 2
**Radiotherapists**						
Mean time	09:54	09:38	06:53	08:54	13:26	14:57
SD	01:35	01:58	01:36	02:07	02:16	01:08
Minimum	07:37	07:30	04:45	05:15	10:46	13:30
Maximum	12:00	11:50	09:04	11:30	17:00	16:40

**Radiation oncologists**						
Mean time	12:35	12:05	07:46	08:52	14:48	15:38
SD	02:37	03:57	03:06	01:59	05:07	06:57
Minimum	10:00	06:00	05:00	06:00	08:15	08:45
Maximum	16:59	15:56	12:33	11:00	20:14	23:14

**Whole group**						
Mean time	11:07	10:45	07:19	08:53	14:04	15:16
SD	02:27	03:08	02:24	01:57	03:41	04:29
Minimum	07:37	06:00	04:45	05:15	08:15	08:45
Maximum	16:59	15:56	12:33	11:30	20:14	23:14

*p* value	0.055	0.201	0.873	0.936	0.584	0.584

Abbreviations: LN, lymph node; SD, standard deviation.

**Fig. 3:**
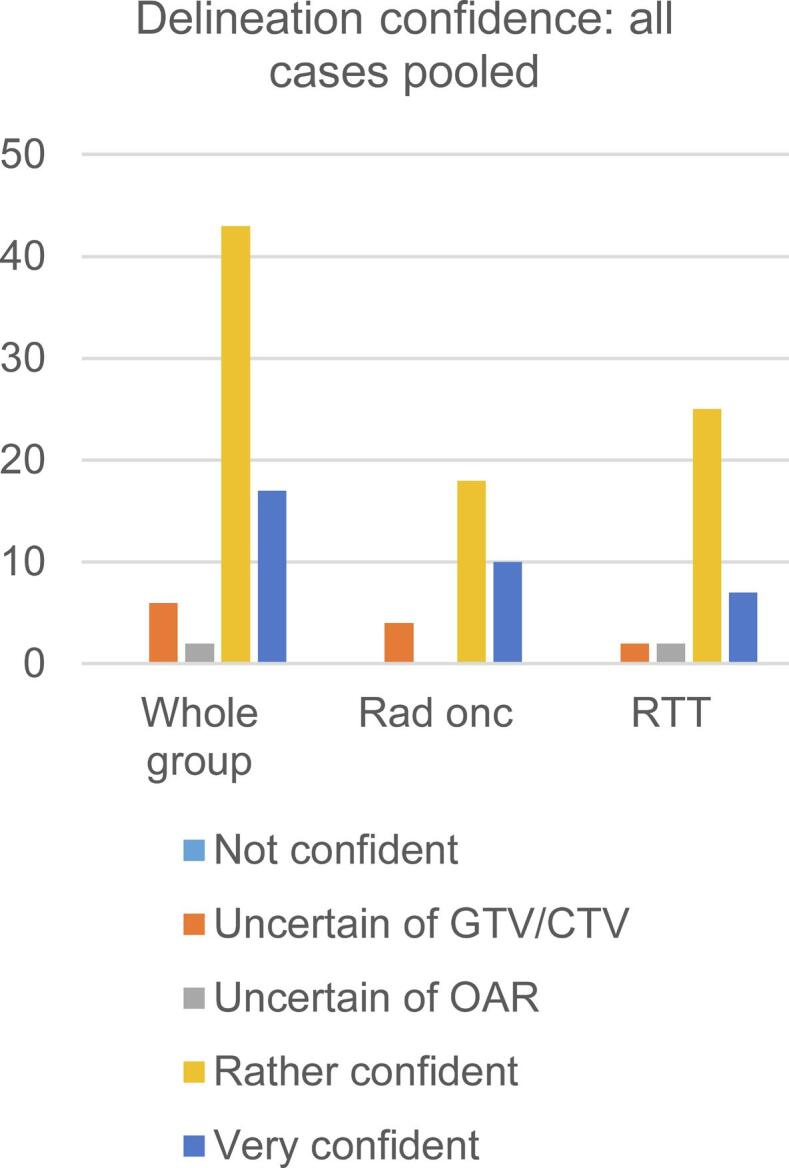
Histogram displaying the confidence of contour adaptation indicated by all observers for all 6 cases pooled, with number of observers on the y-axis. Rad onc, radiation oncologist; RTT, radiation therapist; GTV, Gross Tumor Volume; CTV, Clinical Target Volume; OAR, organs at risk.

Fig. 3 illustrates confidence in the delineation process for all cases pooled and for individual cases (Supplementary Fig. C). Overall, both RTTs and ROs felt confident about their delineations and the option ‘not confident’ was never chosen. However, one RO didn’t delineate prostate cancer cases and another RO didn’t delineate rectal cancer cases, because they did not feel competent to do so. No significant association was found between delineation confidence and conformity.

Concerning treatment planning, the 12 plans of the smallest and largest target volumes per case all were clinically acceptable with adequate target coverage and compliance with constraints for OAR. For example in the prostate cancer cases, the aimed PTV coverage of V3444 cGy > 99 % was reached in all cases with coverage differing with 0.63 Gy at most. The OAR constraints (rectum D1cc < 3800 cGy and bladder D5cc < 3700 cGy) were met with a maximum difference of 0.27 Gy for rectum D1cc and 2.77 Gy for bladder D5cc. For the rectal cancer cases, differences in coverage were very limited.

## Discussion

To increase the efficiency of online adaptive radiotherapy workflows, RTTs are increasingly trained to take the lead for the different parts involved. Within this study we evaluated differences in GTV/CTV contour adaptations performed by ROs and RTTs for patients with LN-oligometastases, rectal- and prostate cancer in a simulated oMRgRT ATS workflow environment, and compared them with the contours used for the respective clinical MR-Linac treatments.

With mean DSC, CI and generalized CI-values in the range of 0.81–0.94, 0.87–0.95 and 0.63–0.85 for prostate cancer, rectal cancer and LN-oligometastases, respectively, we can conclude that the conformity of contouring was overall good and acceptable. Apart from a marginally significant difference in one prostate cancer case, no significant differences were found in DSC and CI between RTTs and ROs. Despite differences in contouring experience (reflecting clinical practice), significant differences were not observed. The lower conformity values for LN targets and larger spread of the IQR are most likely due to the smaller volumes of these targets: small delineation adjustments can lead to relatively larger differences compared to the other sites with considerably larger volumes. Furthermore, the identified differences did not result in unacceptable violations of treatment plan parameters, with adequate target coverage and OAR doses not exceeding clinical constraints for all cases. This is in line with the experience published by Hellebust et al. for expert consensus contours in a setting of MRI-based delineation for cervical cancer brachytherapy [20].

Time needed for delineation was similar for RTTs and ROs with an increase in delineation time being associated with slightly higher conformity, as expected. Mean delineation time was 7–15 min including OAR adaptations with a maximum of 23 min, all not exceeding a workable timeframe for an online treatment setting. However, extra delineation time bares the risk of movement and deformation of targets or OAR and therefore the delineation process should balance between time needed and precision intended.

At University Medical Center Utrecht in 2019 the RTT-led ATS workflow on the MR-Linac was implemented after having developed an in-house training program including online target and OAR contouring and treatment planning. An increasing proportion of our RTTs is becoming MR-Linac competent, while several have gained a ‘super specialization’ in this field. Fully RTT-led MR-Linac treatments are currently the routine practice for the treatment of oligo LN-oligometastases, prostate- and rectal cancer, allowing for a more efficient workflow and scheduling of patients. ROs and MPs are always available on demand, but their availability is no longer a limiting factor. Despite the shift in task allocations, the primary responsibility for quality patient care stays with the treating clinician. Treatment time slots have been reduced, helping to increase the daily treatment capacity of the MR-Linacs. For more complex tumor sites ROs are still onsite for online contouring, but the overall contouring load is gradually shifting to RTTs.

Previous studies report on the capability of trained RTTs for delineating radiation targets based on standardized contouring protocols [7], [21], [22]. The latter study describes interobserver variability of prostate delineations on CT and MRI between RTTs and ROs. Although these studies have been performed in an offline pretreatment setting, the relevance within the context of oMRgRT in prostate cancer is mentioned, and their study design resembles ours. Median DSC of 0.93–0.96 were found, similar to our mean DSC of 0.91–0.94 with regard to RTT data. Delineation time on MRI was also comparable (median of 9.6–9.8 and mean of 9.4–9.5 min respectively).

Regarding an MR-Linac ATS workflow, Willigenburg et al. analyzed RTT-derived prostate CTV and OAR delineations [13]. Different to our study, online contour adaptations performed by RTTs were retrospectively judged by two ROs and adjusted when necessary, yielding a median DSC of even 0.99–1.00. Direct comparisons with our results are difficult due to the different study design, but comparably demonstrate a high level of conformity.

Since the introduction of oMRgRT, some studies described the important role of RTTs for online treatment approaches [11], [23], [24]. Hales et al. described a novel ‘clinician-lite’ prostate treatment adapt-to-position (ATP) MR-Linac workflow, with a prominent role for RTTs [23]. As an ATP workflow does not involve recontouring, contour adjustment comparisons are not applicable, however the potential role for RTTs in target recontouring in ATS workflows is described, with adequate education and training as a prerequisite [23]. Another study found DSC-values over 0.96 for OAR recontouring by two ROs, three RTTs and one research fellow in 10 patients with pelvic tumor sites. Subsequent non-physician treatment plans were acceptable in only 91.25 %, but in 97.2 % if one complex case was excluded, leading to the conclusion that the most complex oMRgRT treatments might be excepted for RTT-led workflows [24].

Also, adequate education and training of staff before implementing (innovations regarding) online MR-guided adaptations is of importance. Apart from training in MR-safety, MR-imaging, image acquisition, processing and interpretation of images and MR-sequences, a proper training for RTTs in target and OAR contour adaptation is essential prior to independently practicing online contour adaptations. RTTs who completed their basic professional training and a specific oMRgRT training can gain competence for online contour adaptations. In our institution, staff is trained according to the tumor site specific workflow. An approach for implementation of oMRgRT in clinical practice and for training of staff, is described in the ESTRO-ACROP recommendations [11]. Since both the national professional education for RTTs, ROs and MPs and the allocation of roles in a clinical workflow differs between countries and institutions [11], [23], different strategies might be chosen regarding the division of tasks and responsibilities in relation to oMRgRT.

A possible limitation of the current study is that we restricted this effort to 3 different treatment indications on the MR-Linac, while more tumor sites are currently treated clinically. Applicability of conformity related findings is therefore limited to these 3 treatment indications. The chosen treatment indications however, currently represent the majority of treated patients on our MR-Linac. As of yet, for other indications ROs are still in the lead for online adaptions. The number of included cases was limited, but on the other hand a group of 12 observers can be considered quite large. Furthermore, we present our institutional experiences and realize that other centers might work with different workflows, logistics or task allocations or have yet to start with MR-guided radiotherapy. Nevertheless, our practices might serve as an example for other centers that aspire to increase the involvement of RTTs in oMRgRT. Strengths of the current study include the use of multiple measuring techniques of conformity, analyses of both delineation time and confidence in the delineation process and the finding that despite some contouring variations, all treatment plans were acceptable.

### Conclusions

To conclude, contour adaptations as needed in adaptive online radiotherapy workflows can accurately be performed by RTTs for prostate cancer, rectal cancer and LN-oligometastases, after dedicated training. Conformity of the derived contours is high and comparable to contour adaptations as provided by ROs. Increasing use of oMRgRT and expected accompanying future developments including gating and tracking and artificial intelligence supported steps in the treatment process create potential for expanding the role of RTTs.

## Funding

This research did not receive any specific grant from funding agencies in the public, commercial, or not-for-profit sectors.

The authors confirm that written informed consent has been obtained from the involved patients or if appropriate from the parent, guardian, power of attorney of the involved patients; and, they have given approval for this information to be published in this case series.

## Declaration of Competing Interest

The authors declare that they have no known competing financial interests or personal relationships that could have appeared to influence the work reported in this paper.

## Figures and Tables

**Supplementary Fig. C.1:**
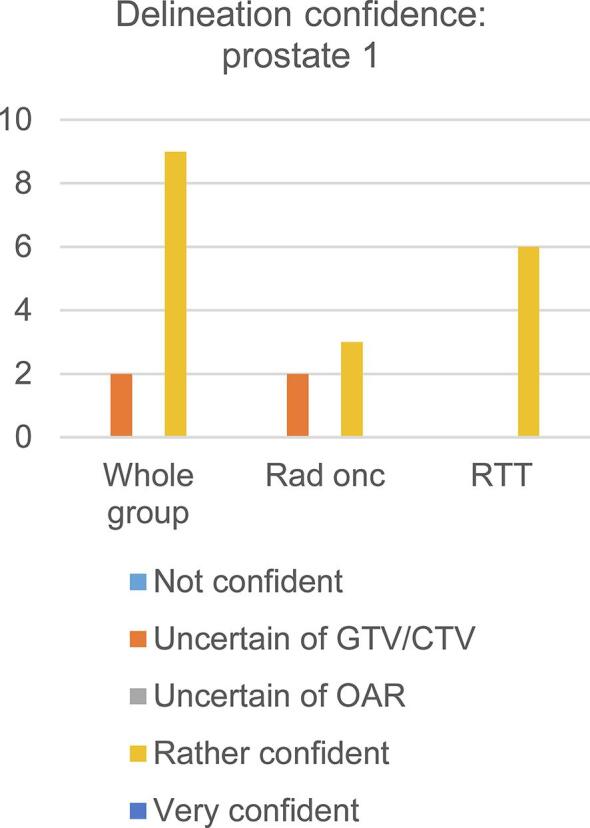
Histograms displaying the subjectively indicated feeling of confidence concerning the delineation per case: prostate 1 (1), prostate 2 (2), lymph node single (3), lymph node multiple (4), rectum 1 (5), rectum 2 (6), with number of delineators on the y-axis. Abbreviations: Rad onc, radiation oncologist; RTT, radiation therapist; GTV, Gross Tumor Volume; CTV, Clinical Target Volume; OAR, organs at risk.

**Supplementary Fig. C.2:**
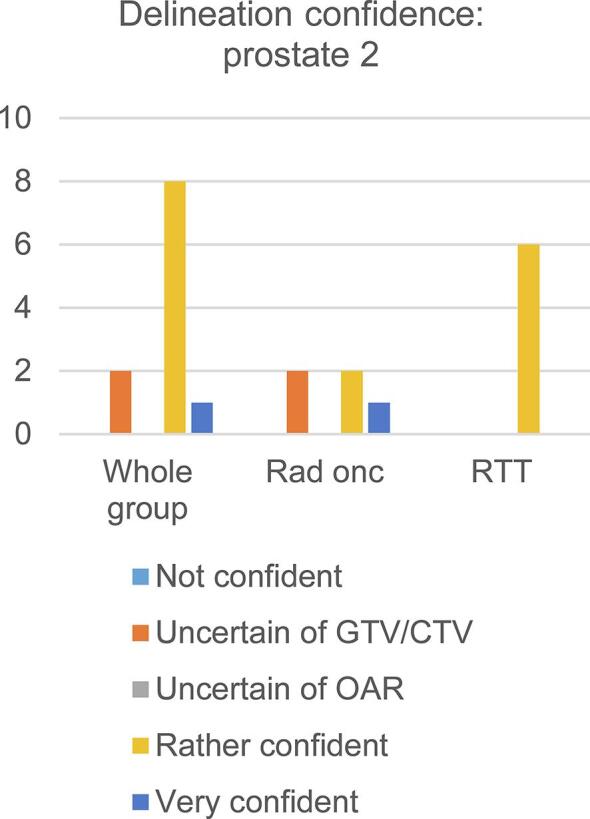
Histograms displaying the subjectively indicated feeling of confidence concerning the delineation per case: prostate 1 (1), prostate 2 (2), lymph node single (3), lymph node multiple (4), rectum 1 (5), rectum 2 (6), with number of delineators on the y-axis. Abbreviations: Rad onc, radiation oncologist; RTT, radiation therapist; GTV, Gross Tumor Volume; CTV, Clinical Target Volume; OAR, organs at risk.

**Supplementary Fig. C.3:**
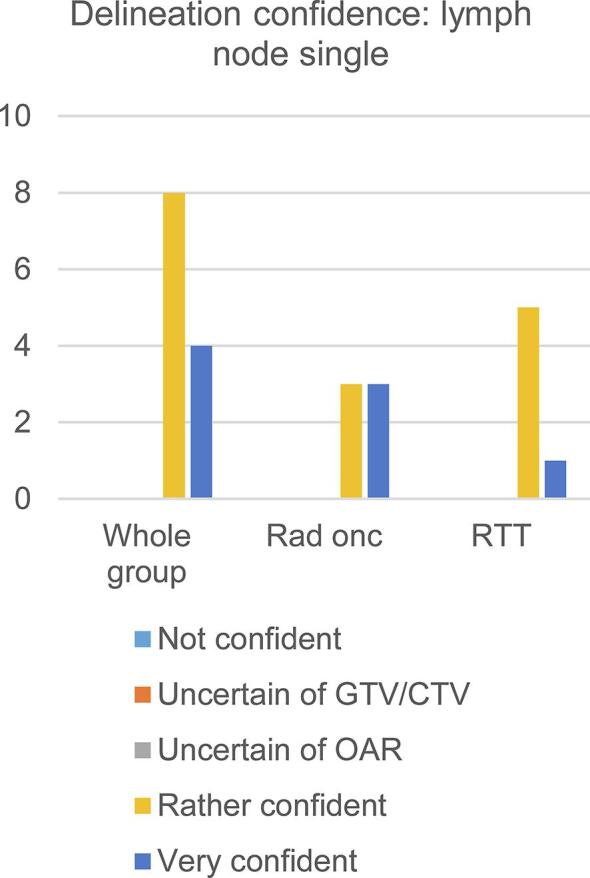
Histograms displaying the subjectively indicated feeling of confidence concerning the delineation per case: prostate 1 (1), prostate 2 (2), lymph node single (3), lymph node multiple (4), rectum 1 (5), rectum 2 (6), with number of delineators on the y-axis. Abbreviations: Rad onc, radiation oncologist; RTT, radiation therapist; GTV, Gross Tumor Volume; CTV, Clinical Target Volume; OAR, organs at risk.

**Supplementary Fig. C.4:**
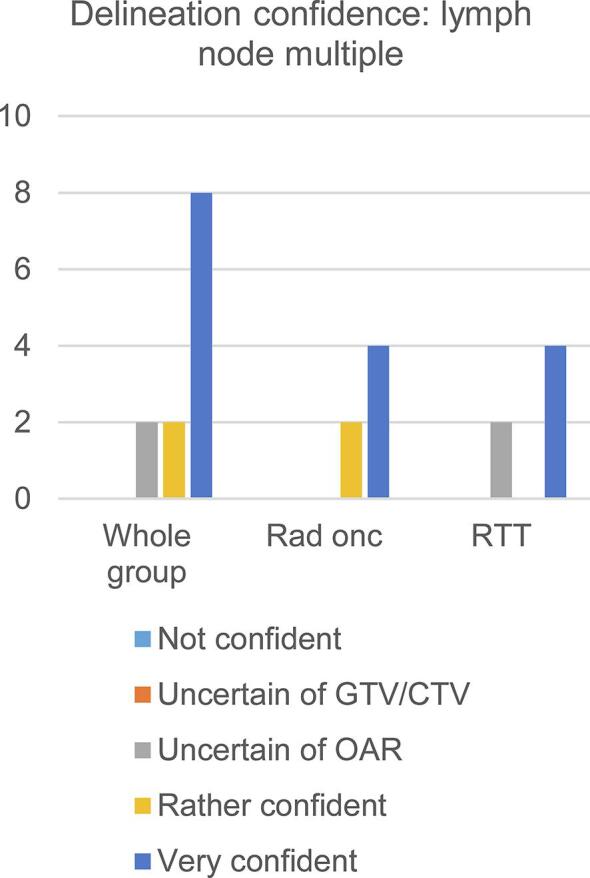
Histograms displaying the subjectively indicated feeling of confidence concerning the delineation per case: prostate 1 (1), prostate 2 (2), lymph node single (3), lymph node multiple (4), rectum 1 (5), rectum 2 (6), with number of delineators on the y-axis. Abbreviations: Rad onc, radiation oncologist; RTT, radiation therapist; GTV, Gross Tumor Volume; CTV, Clinical Target Volume; OAR, organs at risk.

**Supplementary Fig. C.5:**
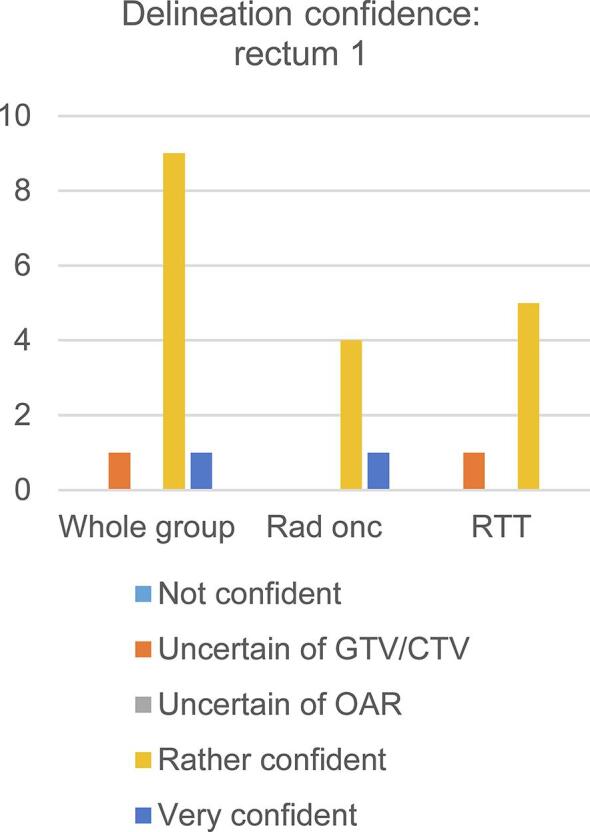
Histograms displaying the subjectively indicated feeling of confidence concerning the delineation per case: prostate 1 (1), prostate 2 (2), lymph node single (3), lymph node multiple (4), rectum 1 (5), rectum 2 (6), with number of delineators on the y-axis. Abbreviations: Rad onc, radiation oncologist; RTT, radiation therapist; GTV, Gross Tumor Volume; CTV, Clinical Target Volume; OAR, organs at risk.

**Supplementary Fig. C.6:**
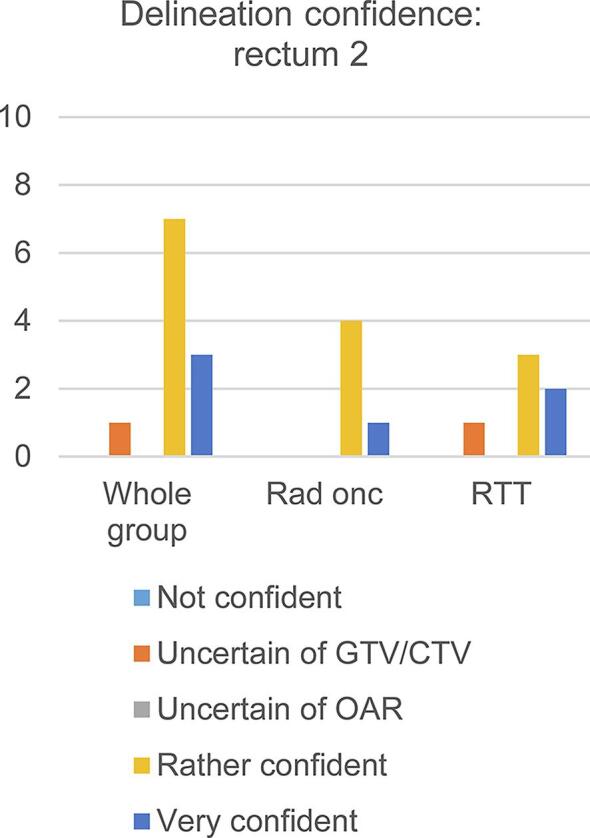
Histograms displaying the subjectively indicated feeling of confidence concerning the delineation per case: prostate 1 (1), prostate 2 (2), lymph node single (3), lymph node multiple (4), rectum 1 (5), rectum 2 (6), with number of delineators on the y-axis. Abbreviations: Rad onc, radiation oncologist; RTT, radiation therapist; GTV, Gross Tumor Volume; CTV, Clinical Target Volume; OAR, organs at risk.

## Appendix A Supplementary material

Supplementary Fig. A.1 Countmaps of lymph node (1) and rectum (2) contour adaptations to illustrate interrater conformity in an axial view (upper contour), coronal view (lower left contour) and sagittal view (lower right contour). Lymph node: voxel size: 0.64 x 0.64 x 2 mm. Rectum: voxel size 0.62 x 0.62 x 2 mm. The colors represent interrater agreement per voxel. Lymph node: dark red = 12 delineators, bright orange = 10 delineators, yellow = 7 delineators, light blue = 5 delineators, dark blue = 0 delineators. Rectum: dark red = 11 delineators, bright orange = 9 delineators, yellow = 7 delineators, light blue = 4 delineators, dark blue = 0 delineators.

Supplementary Fig. A.2 Countmaps of lymph node (1) and rectum (2) contour adaptations to illustrate interrater conformity in an axial view (upper contour), coronal view (lower left contour) and sagittal view (lower right contour). Lymph node: voxel size: 0.64 x 0.64 x 2 mm. Rectum: voxel size 0.62 x 0.62 x 2 mm. The colors represent interrater agreement per voxel. Lymph node: dark red = 12 delineators, bright orange = 10 delineators, yellow = 7 delineators, light blue = 5 delineators, dark blue = 0 delineators. Rectum: dark red = 11 delineators, bright orange = 9 delineators, yellow = 7 delineators, light blue = 4 delineators, dark blue = 0 delineators.

Supplementary Fig. B Scatterplot displaying the correlation coefficient of the relation between conformity index of GTV/CTV (cases pooled except for multiple lymph node case), stated as increase in conformity per extra second of delineation time. On the x-axis delineation time is displayed in minutes.
