# Supplementary figures and images for: Online adaptive MR-guided radiotherapy: Conformity of contour adaptation for prostate cancer, rectal cancer and lymph node oligometastases among radiation therapists and radiation oncologists
Source: Tech Innov Patient Support Radiat Oncol. 2022 Aug 24;23:33–40. doi: 10.1016/j.tipsro.2022.08.004 (PMC9460551; doi:10.1016/j.tipsro.2022.08.004)

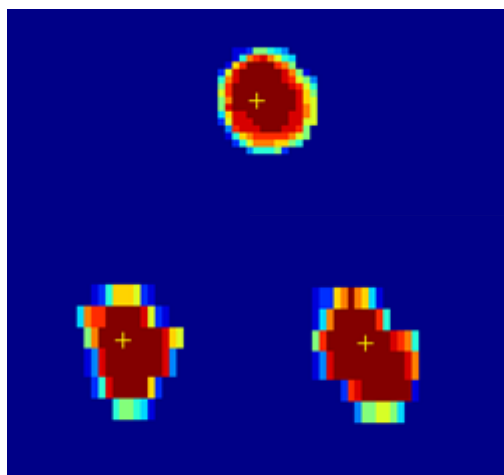

Supplement: Supplementary Fig. A.1 — Countmaps of lymph node (1) and rectum (2) contour adaptations to illustrate interrater conformity in an axial view (upper contour), coronal view (lower left contour) and sagittal view (lower right contour). Lymph node: voxel size: 0.64 x 0.64 x 2 mm. Rectum: voxel size 0.62 x 0.62 x 2 mm. The colors represent interrater agreement per voxel. Lymph node: dark red = 12 delineators, bright orange = 10 delineators, yellow = 7 delineators, light blue = 5 delineators, dark blue = 0 delineators. Rectum: dark red = 11 delineators, bright orange = 9 delineators, yellow = 7 delineators, light blue = 4 delineators, dark blue = 0 delineators. [file mmc1.pdf]

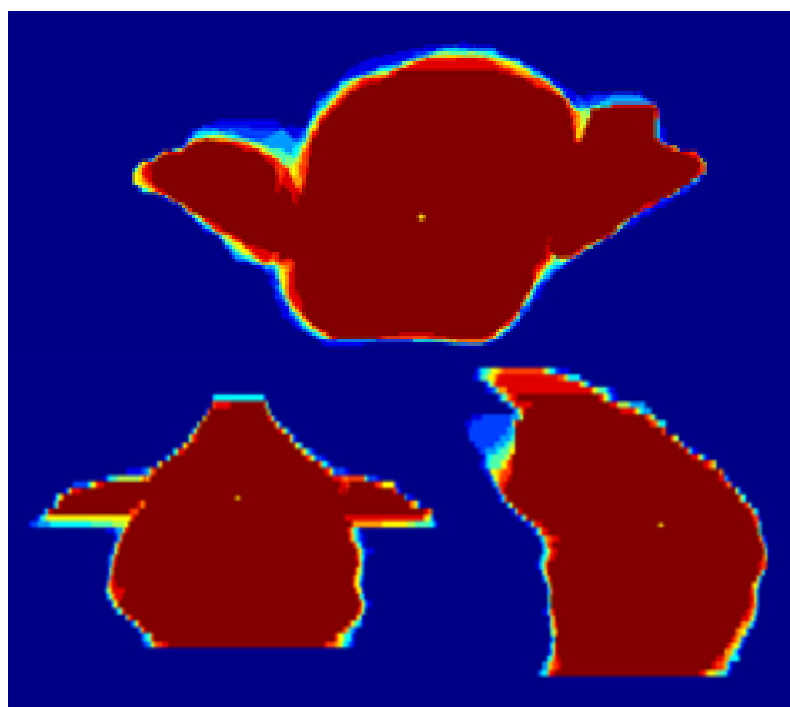

Supplement: Supplementary Fig. A.2 — Countmaps of lymph node (1) and rectum (2) contour adaptations to illustrate interrater conformity in an axial view (upper contour), coronal view (lower left contour) and sagittal view (lower right contour). Lymph node: voxel size: 0.64 x 0.64 x 2 mm. Rectum: voxel size 0.62 x 0.62 x 2 mm. The colors represent interrater agreement per voxel. Lymph node: dark red = 12 delineators, bright orange = 10 delineators, yellow = 7 delineators, light blue = 5 delineators, dark blue = 0 delineators. Rectum: dark red = 11 delineators, bright orange = 9 delineators, yellow = 7 delineators, light blue = 4 delineators, dark blue = 0 delineators. [file mmc2.pdf]

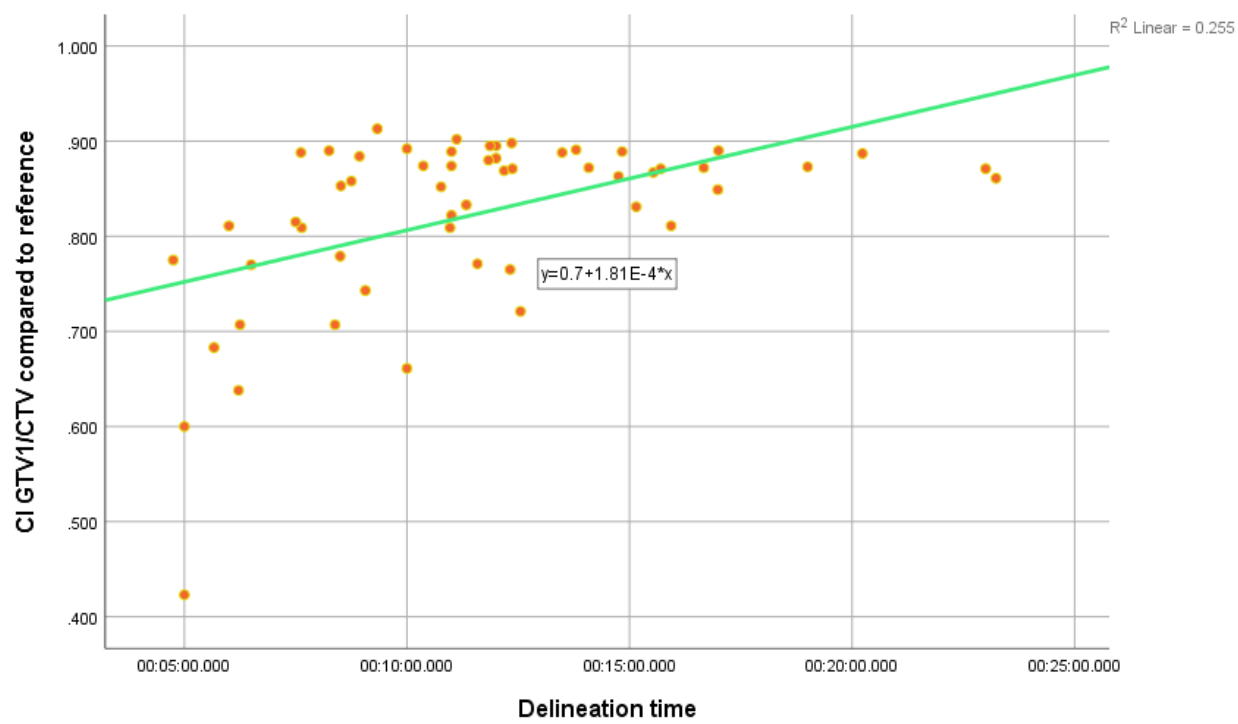

Supplement: Supplementary Fig. B — Scatterplot displaying the correlation coefficient of the relation between conformity index of GTV/CTV (cases pooled except for multiple lymph node case), stated as increase in conformity per extra second of delineation time. On the x-axis delineation time is displayed in minutes. [file mmc3.pdf]
