# Supplementary material for: Online adaptive MR-guided radiotherapy: Conformity of contour adaptation for prostate cancer, rectal cancer and lymph node oligometastases among radiation therapists and radiation oncologists
Source: Tech Innov Patient Support Radiat Oncol. 2022 Aug 24;23:33–40. doi: 10.1016/j.tipsro.2022.08.004 (PMC9460551; doi:10.1016/j.tipsro.2022.08.004)
Supplement: Supplementary data 4 [file mmc4.docx]

**SUPPLEMENTARY TABLES**

A

| Overview of lectures and trainings for RTTs | |
| --- | --- |
| **Title of presentation** | **Duration (minutes)** |
| Introduction | 30 |
| MR-Linac 1998-now | 30 |
| MR-guided/MR-Linac treatment | 30 |
| MR-Linac collaborations physicians/physicists/RTTs | 30 |
| Workflow, processes | 60 |
| MR-Linac hardware | 150 |
| Tour on MR-Linacs by linear accelerator technician | 30 |
| Treatment planning theory | 105 |
| Offline and online planning scenarios MR-Linac | 90 |
| Workshop Monaco (planning system) | 120 |
| MRI theory (image acquisition) | 105 |
| MR safety | 90 |
| MR safety practical training | 60 |
| MR-Linac safety and evacuation training | 120 |
| Image processing theory / clinical practice | 45 |
| Registration / contouring | 60 |
| Auto-contouring | 30 |
| MR-Linac specific RTT function profiles | 30 |
| Image processing in offline and online MR-Linac workflow | 60 |
| First in man, future perspectives | 30 |
| Consortium & Treatment sites for MR-Linac Utrecht | 60 |
| MRI theory | 105 |
| MRI-SIM and MR-Linac | 90 |
| MRI user interface training | 150 |
| Offline contouring training: 3 delineations per tumor site*^#^ | N/A |
| Online contouring training: 15 delineations per tumor site*^#^ | N/A |
| *: Including supervision by RO. #: Duration of contouring training was variable, depending on experience and tumor site. | |

B

| Relation between delineation time and conformity of GTV or CTV. Coefficient (*p* value) | | | | | | |
| --- | --- | --- | --- | --- | --- | --- |
|  | **Whole group** | | **Radiotherapist** | | **Radiation oncologist** | |
|  | **DSC** | **CI** | **DSC** | **CI** | **DSC** | **CI** |
| **1: Delineation time^a^** | | | | | | |
| Prostate 1 | -0.003 (0.295) | -0.006 (0.283) | 0.000 (0.872) | 0.001 (0.795) | -0.001 (0.887) | - 0.002 (0.858) |
| Prostate 2 | 0.000 (0.991) | 0.000 (0.987) | 0.004 (0.336) | 0.007 (0.333) | 0.000 (0.883) | 0.001 (0.880) |
| LN 1 | 0.011 (0.264) | 0.014 (0.277) | 0.004 (0.759) | 0.006 (0.756) | 0.018 (0.193) | 0.023 (0.173) |
| LN 2  GTV 1  GTV 2 | 0.002 (0.876)  0.003 (0.836) | 0.004 (0.824)  0.004 (0.813) | -0.002 (0.942)  -0.005 (0.840) | -0.002 (0.965)  -0.006 (0.862) | 0.007 (0.353)  0.012 (0.378) | 0.010 (0.346)  0.016 (0.399) |
| Rectum 1 | -0.000 (0.860) | -0.000 (0.847) | 0.002 (0.355) | 0.003 (0.383) | -0.001 (0.408) | -0.001 (0.427) |
| Rectum 2 | -0.000 (0.755) | -0.000 (0.818) | -0.002 (0.392) | -0.003 (0.372) | -0.000 (0.962) | 0.000 (0.956) |
| Cases pooled  GTV/CTV^b^ | 0.007 (0.000*) | 0.011 (0.000*) | 0.008 (0.000*) | 0.013 (0.000*) | 0.008 (0.005*) | 0.012 (0.004*) |
| *: Significant difference (*p*<0.05). Abbreviations: GTV, Gross Tumor Volume; CTV, Clinical Target Volume; DSC, Dice similarity coefficient; CI, conformity index. a: Coefficients are stated, as increase or decrease in conformity per minute. b: All cases pooled except for multiple lymph node case. | | | | | | |
